# Antioxidant Defenses in Plants: A Dated Topic of Current Interest

**DOI:** 10.3390/antiox10060855

**Published:** 2021-05-27

**Authors:** Lucia Guidi, Massimiliano Tattini

**Affiliations:** 1 Department of Agriculture, Food and Environment, University of Pisa, Via del Borghetto 80, I-56124 Pisa, Italy; lucia.guidi@unipi.it; 2 Institute for Sustainable Plant Protection, National Research Council of Italy (CNR), Via Madonna del Piano 10, Sesto F.no, I-50019 Florence, Italy

Plants have been challenged against oxidative stress since their appearance on land [1], and their ability not only to survive but to profitably grow in an ever-changing environment allowed for the conquest of land [2,3]. The evolution of an effective antioxidant defense network (*sensu* Mittler [4]) has been of key significance for increasing the complexity of land plants [5], in turn allowing for conquest habitats of increasing stress severity [6]. Indeed, current-day land plants are equipped with an arsenal of “primary” and “secondary” antioxidant defenses to finely tune the levels of different reactive oxygen species (ROS) and to effectively acclimate/adapt to a wide array of environmental stressors of different origins [7]. Plants, as a consequence of their sessile nature, have been capable not only of developing an integrated and modular network to effectively reduce ROS within a sub-lethal concentration range, but also of using ROS as well as changes in redox homeostasis as signaling events to activate further defenses against unpredictable oxidative stress events [8,9,10]. This also conforms to most recent views that “ROS are good” [4]. This is the case, for instance, of low molecular weight antioxidants such as ascorbate and glutathione: they provide essential information on the cellular redox state, and they influence gene expression associated with biotic and abiotic stress responses to maximize defense [11]. Further, plants have used and still use some classes of secondary metabolites, the biosynthesis of which arose during the water-to-land transition of plants, to regulate/modulate ABA and auxin signaling, acting on downstream agents (e.g., H_2_O_2_ and MAPK) [12,13] of the hormone signaling networks [14,15,16,17,18]. There is intriguing, compelling evidence that transcription factors regulating the biosynthesis of these specialized metabolites are under redox control [19,20]. Consequently, changes in cellular redox homeostasis may activate the biosynthesis of specialized metabolites, which, in turn, offer additional redox modulation, therefore constituting a regulatory circuit of hormone/ROS signaling [14,15]. This is consistent with a model for redox homeostasis in which ROS–antioxidant interactions represent metabolic interfaces for signals derived from metabolism and from the environment, as suggested by Foyer and Noctor [11]. It is not surprising that how plants adjust the suite of antioxidant defenses in response to the wide range of environmental stressors has been and still is one of the timely issues in plant science [21,22,23,24,25,26,27]. Indeed, global change imposes the well-known acclimation/adaptation syndrome of living organisms, which may be particularly severe for plants, which do not possess the ‘flight’ strategy usually displayed by other organisms [28]. 

The special issue “Antioxidant defenses in plants” is therefore timely and offers contributions that may help readers to focus on most recent aspects of how plants cope with oxidative stress of different origins. As outlined above, our special issue is not centered merely in exploring the antioxidant that arsenal plants activate to limit oxidative stress and damage, but it also focuses on the signaling/functional roles of ROS (and reactive nitrogen species, RNS as well) in response to “environmental stressors” of different origins. 

This is exactly the case of the review article by Hasanuzzaman and colleagues [29]. The authors provide a comprehensive review of the chemistry of ROS, their generation and subcellular compartmentation. The authors offer a critical review of both the oxidative stress generated and antioxidant defense activated in response to a wide array of stress agents, including soil salinity, water deficit, metal ions excess, and high and low temperatures. Hasanuzzaman and colleagues [29] also critically explore the role of ROS, especially H_2_O_2_ as a signaling molecule, which leads to acclimation processes and confers tolerance under environmental stressors of different origins [27,30,31]. This is consistent with a recent observation that the exogenous application of H_2_O_2_ increases tolerance to abiotic stresses [32]. The authors also focus their attention on the crosstalk between ROS and a range of other reactive species, namely reactive nitrogen (RNS), sulphur (RSS) and carbonyl species (RCS), which might act in concert in signal transduction pathways under stressful conditions [33]. 

The research article by Van Rensburg and Van den Ende [34] is centered on the role of ROS in plants exposed to biotic stresses, and they specifically examine the role of ROS in the responses of *Arabidopsis thaliana* to pathogen infection, namely to *Botrytis cinerea*. Actually, the authors explore how γ-aminobutyric acid (GABA) treatment affects plants challenged against pathogens by modulating ROS homeostasis, a timely issue in plant response to biotic stressors [35]. In a very low concentration range, GABA effectively primed resistance against *B. cinerea*, thereby supporting the author’s idea that GABA signaling was at play, rather than metabolic effects. The view of GABA behaving as a signaling molecule is consistent with its putative role in cellular ROS dynamics. This has a consequential direct impact on plant resistance, since ROS are crucial components of the infection strategy of *B. cinerea*. Of interest is the finding that pretreatment with GABA keeps low H_2_O_2_ levels upon infection, because of enhanced activities of both catalase and glutathione peroxidase, therefore slowing down the infection process [36]. Finally, the authors offer the intriguing hypothesis that GABA signaling might be translated to distal tissues and organs through its constant secretion/uptake between cells (as also occurs in animals) [37,38]: the issue merits further investigation, as it might be involved in systemic acquired resistance [39].

The role of reactive species, namely of RNS, in plant responses to biotic stressors is also the focus of the research paper by Labudda and colleagues [40]. The authors explore the interplay between RNS involved in signal transduction and plant defense against the cyst nematode *Heterodera schachtii* Schmidt’s infection on *Arabidopsis thaliana* roots. Labudda and colleagues [40] provide conclusive evidence for RNS production in root cells as a response to cyst nematode attack and parasitism. In turn, RNS, mostly NO, participate in the regulation of the MAPKs (mitogen activated protein kinase) cascade [41]. The authors also hypothesize that H_2_O_2_, which is known to activate MAPKs [42,43] (including MAPK6), may have been responsible for the intense NO burst and the downstream signaling pathway in *A. thaliana* roots upon *H. schachtii* infestation. The crosstalk between ROS and RNS is crucial in activating defense responses [44], and Lubudda and colleagues [40] report on the potential interplay between the superoxide anion, generated by plasma membrane NADPH oxidase, and NO. They suggest that the formation of peroxinitrite (ONOO-) through the reaction of O_2_- and NO is central to the response of *A. thaliana* roots to infection, since ONOO- may freely pass cell membranes and interacts with bio-targets [45]. Therefore, the findings of Labudda et al. [40] offer further support about the central role of NO in root-pathogen interaction, which may be significant to developing novel plant-protection strategies [46].

The roles of reactive oxygen and nitrogen species in plants suffering from abiotic stress agents, ranging from salt/drought stress to high light and nutrient deficiency, have also been indirectly investigated in the review article by Pardo–Hernández and colleagues [47]. In detail, the authors critically review the pertinent literature on the application of melatonin (MET), a tryptophan derivative that has great success, in stress mitigation [48,49]. MET is a key molecule that is functional in regulating the growth, development and reproduction of plants [50]. This mainly results from MET being involved in the regulation of oxidative stress through its effect not only on the network of enzymatic and nonenzymatic defenses but also on the oxidative signaling driven by both ROS and NO [49,51]. There is indeed evidence of MET being involved in the NO-signaling pathway: for instance, NO-induced S-nitrosylation of antioxidant-related proteins [52] is crucial in regulating the antioxidant capacity of the ascorbic acid/glutathione cycle under nitro-oxidative conditions. Nonetheless, Pardo–Hernandez and colleagues [47] conclude that the mechanisms through which MET and NO crosstalk in ameliorating “plant performance” under stressful conditions is far from being conclusively addressed [41]. 

The ROS- and NO-induced regulation/modulation of gene expression is also the issue investigated by Pucciariello and Perata [53] in their review article. This article represents a curiosity in the context, since it explores the apparent contradiction of the presence and the significance of ROS as well as of RNS (i.e., NO) under low O_2_ availability [54,55,56]. The authors conduct a comprehensive analysis of studies aimed at unveiling how the availability of ROS and NO, and the consequential adjustments in the antioxidant system, allow for plant adaptation under different levels of O_2_ availability and water submergence. Pucciariello and Perata [53] conduct their analysis mainly, but not exclusively, by looking at the molecular events that are responsible for the ROS and NO modulation of the expression of specific transcription factors, such as the group VII ethylene responsive factors (ERF-VII), regulating key developmental processes under hypoxia [57]. They conclude that ROS might act as an additional signaling mechanism, acting in parallel to direct O_2_ sensing. Pucciariello and Perata [53] also highlight the role of ROS production upon post-submergence. In fact, during submergence, turbid water reduces the access of light to underwater organs, while a loss in root hydraulic conductivity may paradoxically lead to dehydration, both phenomena impeding photosynthesis, thereby leading to ROS generation [58]. There is evidence of a key involvement of primary antioxidants in conferring post-submergence tolerance, though the matter still needs investigation. 

Our special issue includes, as expected, a number of contributions that explore how the antioxidant defense system is at work in plants suffering from a range of stress agents of abiotic origin. These articles are mostly, but not exclusively, concerned with the roles of secondary metabolites in mitigating stress-induced photooxidative damage in a range of plants. 

For instance, in their comprehensive review article, Pollastri and colleagues [59] reason about the “antioxidant” functions of isoprene, a volatile isoprenoid mainly emitted by perennial plants [60,61], the biosynthesis of which is strongly activated both by high temperature and solar irradiance but also because of water deficit [62,63]. The authors report evidence of isoprene being capable of quenching a range of reactive oxygen and nitrogen species, as previously reported in a comprehensive review article by Vickers and colleagues [64]. This suggests a direct antioxidant role for isoprene, but whether isoprene scavenges ROS inside the leaf or in the leaf boundary layer, after exiting leaves through stomata, remains unclear. Pollastri and colleagues [59] report evidence that, with respect to nonemitting plants, isoprene-emitting plants are able to maintain high photosynthetic rates for longer periods when exposed to abiotic stresses, not only because of their ROS quenching capacity but also by improving the thylakoid membrane stability [65,66,67]. In other words, isoprene emitters are well suited to “avoiding”, and not only to countering, photooxidative stress. Further, the authors discuss relatively recent findings showing that isoprene may alter both the proteome and the metabolome in some species, resulting in an enhanced biosynthesis of a range of antioxidants [68,69]. Finally, Pollastri and colleagues [59] discuss recent reports suggesting that isoprene may control the antioxidant system, generally acting as a negative regulator of ROS production in a hormone-like manner. Overall, this is consistent with their hypothesis of isoprene as “a molecule with multiple regulatory functions”, which conforms to the general idea that so-called secondary metabolites, whose biosynthesis is highly consuming for plants in terms of energy and carbon skeletons, serve multiple functional roles in plant-environment interactions. In the vast majority of cases, these functions go beyond the mere ability to quench ROS. 

Agati and colleagues [70] discuss the putative antioxidant functions of a relevant class of metabolites synthesized through a branch-pathway of the general phenylpropanoid biosynthetic pathway, specifically the flavonoids. The effective abilities of flavonoids to serve antioxidant functions in an in planta situation is a long-debated question. There are several reasons that the authors suggest are responsible for this continued discussion. First, early experiments provided erroneous beliefs about flavonoids being optimally suited to absorb UV-B wavelengths, whereas flavonoids have molar extinction coefficient maxima at >330 nm (for the review article, see [71,72]). The view about flavonoids being exclusively located in the vacuoles of epidermal cells [73,74] is equally erroneous: flavonoids accumulate in different leaf tissues and subcellular compartments, including the nucleus, the cytoplasm and the chloroplast envelope [75]. Consequently, flavonoids are optimally suited to encounter and scavenge a range of ROS, including H_2_O_2_ and singlet oxygen, as conclusively shown in recent experiments [16,17,76]. Second, the view about ROS, particularly H_2_O_2_, being “inherently” unable to freely move from its generation sites to other cell organs, where flavonoids mostly accumulate, is erroneous [77,78]. The authors also suggest that the picture representing the complexity of the dynamic cellular organization during the most severe stressful conditions [79,80] needs extensive re-drawing in order to delineate the actual scenario within which biological processes occur. Finally, Agati et al. [70] highlight that an unequivocal definition of antioxidants is mandatory in order to unveil the effective antioxidant capacities of different metabolites, including but not limited to flavonoids. The authors report a few examples of such highly contrasting definitions, ranging from the most wide and hence robust definition given by Gutteridge and Halliwell [81] to the most constraining one proposed by Hernandez et al. [82]. The authors propose to simply define a plant antioxidant in terms of its ability to “counter” oxidative stress and damage, the ability being “quantified” by measurements of “oxidative stress reduction”.

In their research article, Marchica and colleagues [83] also investigate the functional role of phenylpropanoids in the response mechanisms of plants facing abiotic stressors, specifically in response to ozone (O_3_), a tropospheric pollutant with a high oxidative potential [84,85]. The authors conducted an experiment on *Salvia officinalis*, looking at both the molecular events regulating the biosynthesis as well as the functional roles of phenylpropanoids in O_3_-stressed plants. The authors showed that a single pulse of O_3_ was capable of greatly affecting the phenylpropanoid biosynthetic pathway, leading to an enhanced biosynthesis of a range of phenylpropanoids, especially of rosmarinic acid, a caffeic acid ester synthesized from 3,4-dihydroxyphenyllactic acid through the action of rosmarinic acid synthase (RSA). As such, rosmarinic acid (which accounted for approx. 50% of the entire phenylpropanoid pool) may serve effective antioxidant functions in O_3_-treated plants, due to the presence of two catechol groups in the benzene rings. Marchica and colleagues [83] also report on the consistent relationship between RAS activity, which increased upon 5 h of O_3_ fumigation, and the consequential enhanced accumulation of rosmarinic acid, which was observed 24 h after the onset of the O_3_ treatment. The authors’ data are therefore consistent with the general view that the enhanced biosynthesis and accumulation of phenolic compounds may well constitute a “secondary” antioxidant defense system, complementing the activity of “primary” antioxidants to avoid irreversible oxidative injury in plants under O_3_ exposure. 

In their research article, Kim and colleagues [86] provide evidence of carotenoids serving antioxidant functions in sweet potato plants exposed to severe heat stress (plants were indeed exposed for 12 h at 47 °C). The authors generated transgenic sweet potato plants using the Orange gene, encoding for a protein that behaves as a holdase chaperone, a post-transcriptional regulator of phytoene synthase (PSY), a rate-limiting enzyme in the carotenoid biosynthetic pathway [87]. Kim and colleagues [86] show that transgenic plants are more tolerant to heat stress compared to wild-type plants, based on a lower accumulation of both H_2_O_2_ and membrane permeability, which was particularly evident in the plant suffering from the most severe heat stress. The authors attribute the superior heat tolerance to the dramatically higher carotenoid concentration, especially ß-carotene and cryptoxanthin, detected in transgenic plants. This is consistent with the notion of ß-carotene being most suitable to quench singlet oxygen [88], whereas cryptoxanthin (which is generated through the hydroxylation of ß-carotene [89,90]) likely confers a greater stability to thylakoid membranes, in a similar fashion to zeaxanthin [91,92]. 

The increase of the overall antioxidant defense system is of crucial significance to the profitable growth of a range of annual crop species (which do not constitutively possess the antioxidant arsenal of the vast majority of wild plants) that are frequently exposed to severe environmental pressures imposed by global change [62]. Padilla and colleagues [93] explore the effectiveness of vegetable grafting (a sustainable technique commonly used to enhance plant tolerance to abiotic stressors) in enhancing plant tolerance to drought stress [94]. For this purpose, the authors grafted the pepper (*Caspicuum annuum*) landrace “Sueca” on two *C. annuum* hybrids, H90 and H92, which have been previously reported to be sensitive and tolerant to drought stress, respectively. Padilla and colleagues [93] performed measurements on an impressive amount of physiological and biochemical traits to assess the rootstock effect in conferring drought tolerance to a scion/rootstock combination. Actually “Sueca” grafted on H92 displayed a better photosynthetic performance under severe drought, which translated into a lower peroxidation of membrane lipids compared to the Sueca/H90 combination. The two scion/rootstock combinations greatly differed for the biosynthesis and metabolism of ascorbic acid, with Sueca/H92, but not Sueca/H90, showing an increased accumulation of ASA when the drought stress became severe. The authors hypothesize that in addition to well-known ROS scavenging properties of ASA, the ASA-zeaxanthin interaction and the consequential enhancement of nonphotochemical quenching [95,96] were likely responsible for the sustained photosynthesis in Sueca/92 compared to Sueca/90.

This Editorial is in the form of a brief review article. Our efforts have been to provide readers not only with a brief description of each original contribution, but with up-to-date literature concerning the role of oxidative signals as well as of the wide range of “antioxidants” that help plants to cope with environmental stressors of different origins. We hope our efforts will be useful to readers of Antioxidants. 
